# Immunopharmacology of Post-Myocardial Infarction and Heart Failure Medications

**DOI:** 10.3390/jcm7110403

**Published:** 2018-10-31

**Authors:** Mona Panahi, Nimai Vadgama, Mathun Kuganesan, Fu Siong Ng, Susanne Sattler

**Affiliations:** 1 National Heart and Lung Institute, Imperial College London, London W12 0NN, UK; mp4213@ic.ac.uk (M.P.); nv412@imperial.ac.uk (N.V.); f.ng@imperial.ac.uk (F.S.N.); 2 University College London Medical School, University College London, London WC1E 6BT, UK; mathun.kuganesan.17@ucl.ac.uk

**Keywords:** myocardial infarction, heart failure, immunopharmacology, immunomodulation

## Abstract

The immune system responds to acute tissue damage after myocardial infarction (MI) and orchestrates healing and recovery of the heart. However, excessive inflammation may lead to additional tissue damage and fibrosis and exacerbate subsequent functional impairment, leading to heart failure. The appreciation of the immune system as a crucial factor after MI has led to a surge of clinical trials investigating the potential benefits of immunomodulatory agents previously used in hyper-inflammatory conditions, such as autoimmune disease. While the major goal of routine post-MI pharmacotherapy is to support heart function by ensuring appropriate blood pressure and cardiac output to meet the demands of the body, several drug classes also affect a range of immunological pathways and modulate the post-MI immune response, which is crucial to take into account when designing future immunomodulatory trials. This review outlines how routine post-MI pharmacotherapy affects the immune response and may thus influence post-MI outcomes and development towards heart failure. Current key drug classes are discussed, including platelet inhibitors, statins, β-blockers, and renin–angiotensin–aldosterone inhibitors.

## 1. Introduction

Heart failure (HF) defines the inability of the heart to meet the metabolic demands of the body and is a common consequence of myocardial infarction (MI). A recent study shows that 62.7% of patients with a hospital diagnosis of acute MI (AMI) developed HF in the following six years [1]. Local hypoxia after MI causes cardiomyocyte death and the immune system is recruited to remove necrotic debris and initiate scar formation [2]. Severe tissue damage may, however, cause a strong inflammatory response and result in excessive fibrosis, ongoing immune autoreactivity and adverse remodeling towards HF [3]. Preclinical and clinical agents targeting immunological pathways have been explored for post-MI benefits and the most promising candidates include inhibitors of early inflammatory mediators and pro-inflammatory cytokines. In a recent systematic review, we provide a thorough overview of established and novel immunomodulatory treatments post-MI and during HF and show that, despite promising outcomes in selected trials, heterogeneous patient populations and inconsistent trial design complicate analysis. A final conclusion about the true clinical benefit of these novel agents is therefore still lacking [4].

**Figure 1:**
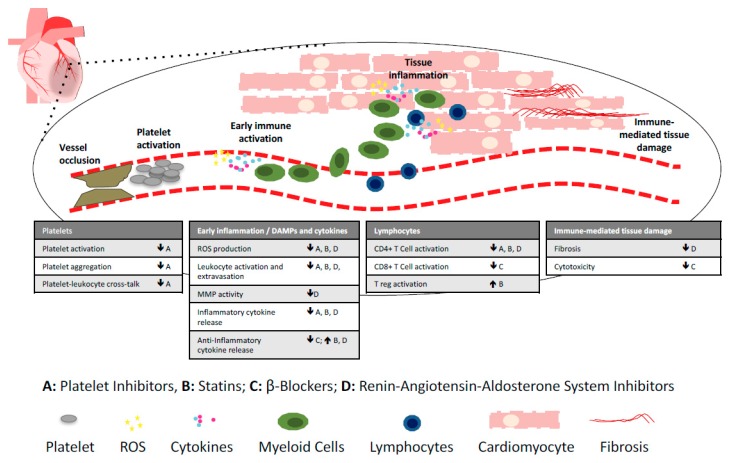
Immunomopharmacology of commonly used post-MI and heart failure drugs. The immune system is involved in all post-MI processes, including platelet activation immediately after vessel occlusion, early immune activation leading to leukocyte extravasation into the heart, and immune-mediated tissue damage. Currently used post-MI drugs with immunomodulatory effects include platelet inhibitors (**A**), statins (**B**), beta-blockers (**C**), and drugs targeting the renin–angiotensin–aldosterone system (**D**), including angiotensin converting enzyme (ACE) inhibitors, angiotensin receptor blockers, angiotensin receptor–neprilysin inhibitors and aldosterone antagonists. DAMPs; danger associated molecular patterns, ROS; reactive oxygen species, MMP; metalloproteinase. ↓ decrease, ↑ increase.

Importantly, standard drugs currently used in the post-MI therapeutic regimen also affect the immune system [5] (Figure 1; Table 1). This needs to be considered carefully when designing future immunomodulatory therapies, and it is feasible that immunomodulation may in fact contribute to the cardioprotective effects of current post-MI therapy.

Here we discuss the immunological effects of routinely prescribed post-MI drugs, including platelet inhibitors, statins, β-blockers, and drugs targeting the renin–angiotensin–aldosterone system, including angiotensin converting enzyme (ACE) inhibitors, angiotensin receptor blockers, angiotensin receptor–neprilysin inhibitors, and aldosterone antagonists. We illustrate that potential interactions of existing and emerging immunomodulatory interventions with routine pharmacotherapy should be considered when designing patient therapeutic regimens.

## 2. Platelet Inhibitors

Antithrombotic drugs are administered post-MI to reduce the risk of thrombus formation, thus decreasing the risk of MI reoccurrence [37]. Pharmacological agents used to achieve this are antiplatelet drugs, anticoagulants, and thrombolytic drugs, with platelet inhibitors being the most prominent antithrombotic drug class in routine long-term use for secondary prevention of MI.

Platelet and monocyte counts increase with MI severity and platelets continue to be activated in HF, irrespective of therapy [38]. However, platelets have important roles beyond haemostasis. They interact with the endothelium and leukocytes to promote activation, adhesion, and extravasation of monocytes, neutrophils, and lymphocytes and thereby contribute not only to thrombotic occlusion and microembolisation of coronary arteries, but also to inflammation [38]. Cyclo-oxygenase (COX) inhibitors (e.g., aspirin) and adenosine diphosphate receptor (P2Y_12_) antagonists (e.g., clopidogrel, ticagrelor, prasugrel) are routinely prescribed platelet inhibitors post-MI [39].

### 2.1. COX Inhibitors

COX inhibition irreversibly blocks platelet aggregation [40]. The most prominent COX inhibitor, aspirin, has been shown to block reactive oxygen species (ROS) formation and platelet and leukocyte activation [6]. Monitoring inflammatory markers of 310 AMI patients in the warfarin–aspirin re-infarction study (WARIS-II, 2003) showed that 160 mg/day of aspirin reduced high-sensitivity C-reactive protein (hs-CRP) at both three months and four years and Interleukin (IL)-6 was also significantly reduced at a four-year follow-up, compared to warfarin alone [7].

### 2.2. P2Y_12_ Antagonists

P2Y_12_ receptors play a primary role in haemostasis and platelet aggregation upon adenosine diphosphate (ADP) binding [41]. Examples of P2Y_12_ inhibitors include clopidogrel, ticagrelor, and prasugrel, which are routinely prescribed post-MI [37]. P2Y_12_ inhibitors can suppress degranulation, platelet–leukocyte aggregate formation, and expression of pro-inflammatory cytokines [6]. In a clinical trial with 120 patients undergoing percutaneous coronary intervention (PCI), both prasugrel and clopidogrel significantly reduced hs-CRP levels [8].

### 2.3. Glycoprotein (GP) IIb/IIIa Inhibitor

The platelet receptor GPIIb/IIIa binds fibrinogen, thus bridging adjacent platelets for aggregation [42]. A GPIIb/IIIa inhibitor, tirofiban, has been shown in a small study by Ercan et al. (2004) to significantly reduce C-reactive protein (CRP) in non-ST elevation MI (NSTEMI) patients when prescribed for 48 h following MI [9].

Due to the various pro-inflammatory effects of activated platelets, their inhibition also suppresses inflammatory chemokine, cytokine, and adhesion molecule expression. Although not their intended primary function, platelet inhibitors may therefore possess potent anti-inflammatory effects.

## 3. Statins

Statins inhibit the liver enzyme β-hydroxy β-methylglutaryl-coenzyme A (HMG-CoA) reductase, which is involved in the production of low-density lipoprotein–cholesterol (LDL–C). Reducing LDL–C levels is anticipated to reduce atherosclerotic plaque formation and protect from re-infarction [43]. Statins are the most commonly prescribed lipid-lowering agent worldwide [44]. However, statins exert additional advantageous cardiovascular effects, which are independent of their lipid-lowering effects, including improvement of endothelial function, antithrombotic effects, antioxidant effects, antiproliferative effects on smooth muscle cells, and anti-inflammatory effects [45,46]. Previous meta-analyses have shown that the use of statins during preloading in patients undergoing PCI significantly reduced the rates of periprocedural MI and Major Adverse Cardiovascular events (MACE) [47,48]. In non-ischaemic HF, atorvastatin significantly improves patients’ left ventricular ejection fraction (LVEF), which is thought to be due to attenuation of adverse left ventricular (LV) modelling [49].

Recent studies have suggested that their favourable clinical outcomes could be in part due to their immunomodulatory properties affecting immune cell proliferation and migration [50]. Statins modulate the T cell repertoire by blocking antigen-specific T cell activation and inducing an anti-inflammatory and regulatory T (Treg) cell phenotype. Porcine studies reported a 25-fold reduction in major histocompatibility (MHC) class II molecule expression on vascular endothelial cells following statin administration [10]. Alongside MHC class II, reduced expression of MHC class I, and co-stimulatory molecules CD28 and CD40 have been described [11]. MHC class II is found constitutively on antigen-presenting cells and is essential for antigen-dependent T cell activation. Multiple cell types, including endothelial cells, monocytes, and macrophages, can be stimulated by interferon (IFN)-γ to express MHC class II molecules on their cell surface to activate the adaptive immune system, but statins can intercept this process [12]. Statins further block intracellular GTPases, which impairs protein antigen uptake and subsequently dampens T cell activation [13].

Statins also directly promote immune tolerance by increasing the expression of transcription factor forkhead box P3 (FoxP3) in Treg cells, along with immune regulatory cytokines IL-10 and tumour growth factor-β (TGF-β) in atherosclerotic plaques [14]. Treg cell migration and differentiation via the C-C motif ligant-1 (CCL1) chemokine is influenced by statin administration [15] and, clinically, an increase in FoxP3 mRNA levels has been observed in male patients following one month of statin use [16]. 

Simvastatin administered to ST-elevation MI (STEMI) patients reduced the levels of the inflammatory cytokines IL-2 and tumour necrosis factor (TNF)-α in a dose-dependent manner, with a significant but transient drop in CRP in the first week, compared to placebo treatments. Although CRP returned to normal by day 30, the flow-mediated dilation of the brachial artery was proportional to the initial statin dose [17]. This study indicates that the timing and dose of statin therapy influence the inflammatory and endothelial responses following MI. 

Considering the detrimental effect of post-MI adaptive immune-autoreactivity and beneficial effect of Treg cells in heart regeneration [51], it is feasible that these striking effects on the adaptive immune system may play an important role in the observed clinical effects of statins in slowing the post-MI progression to heart failure. 

## 4. β-Blockers 

β-Blockers are used post-MI to reduce heart rate and blood pressure (BP). They constitute a heterogeneous group of drugs blocking the adrenoreceptors of the sympathetic nervous system. They can be selective inhibitors of β1-adrenoreceptors (e.g., metoprolol, bisoprolol, low concentrations of nebivolol), nonselective blockers of both β1- and β2-adrenoreceptors (e.g., propranolol, high concentrations of nebivolol), and a third generation of nonselective β-blockers/α1 blockers (e.g., carvedilol). Nebivolol, atenolol, and bisoprolol are the main treatment options after acute coronary syndrome (ACS), largely due to their superior cardioselectivity when compared to other β-blockers [52]. β-Blockers have infarct-reducing properties, improve cardiac function, and can help to prevent future infarcts [53]. Their immunological effects vary and may prove beneficial or detrimental, depending on the exact setting.

### 4.1. Selective β1 Antagonists

Bisoprolol reduces circulating TNF-α levels and helps to restore the dysregulated cytokine network in dilated cardiomyopathy (DCM) patients [18]. Similarly, metoprolol lowers plasma levels of TNF-α, IL-6, IL-10, soluble IL-2 receptor (sIL-2R), monocyte chemoattractant peptide-1 (MCP-1), and IL-8 in chronic HF (CHF) patients [19]. Post-MI, however, metoprolol may in fact attenuate the anti-inflammatory effects of statins, as assessed by CRP level [20]. Metoprolol is available in two formulations, immediate-release metoprolol tartrate (MT) and slow-release metoprolol succinate (MS), of which only MS is recommended for the treatment of heart failure [54].

### 4.2. Non-Selective β-Blockers

Propranolol strongly upregulated IL-1β and IL-6 gene expression in the myocardium 6 h after MI in a rat model [21]. Similar to metoprolol, propranolol may also attenuate the anti-inflammatory effects of statins [20]. However, it is noted that propranolol is thought to increase the activity of NK cells [22], which may modulate the inflammatory milieu in a positive manner [55].

### 4.3. Nonselective β-Blockers/α1 Blockers

Carvedilol reduces the levels of HLA-DR positive lymphocytes and cytotoxic T cells in the blood of CHF patients [23] and is therefore of particular interest. The presence of these cells has been implicated in myocardial dysfunction, cytotoxicity, and increased risk of severe post-MI HF [56]. Carvedilol has also been shown to decrease the production of ROS, such as H_2_O_2_, which is responsible for driving calcium overload in HF [24]. In a study on experimental infection with *Trypanosoma cruzi*, carvedilol decreased CCL2 levels and increased the levels of the anti-inflammatory cytokine IL-10, resulting in decreased inflammatory infiltration in cardiac muscle [25].

Depending on the drug type and timing of administration, β-blockers may thus have strikingly distinct immunological consequences. They might interfere with the resolution of inflammation in AMI, yet the blocking of adaptive autoreactivity during CHF may be a significant positive contributor to the beneficial effects of β-blockers in these patients.

## 5. Drugs Targeting the Renin–Angiotensin–Aldosterone System

The Renin–Angiotensin–Aldosterone System (RAAS) is involved in regulating BP, blood volume, and sodium concentration. In response to a BP drop, the kidneys release renin, which cleaves angiotensinogen into angiotensin I. The angiotensin converting enzyme (ACE) then converts angiotensin I into angiotensin II (ATII). ATII binds to AT_1_ receptors, causing systemic vasoconstriction, resulting in increased systemic arterial pressure and restoration of BP. ATII thus stimulates cardiac hypertrophy and myocardial fibrosis [57]. Notably, ATII signalling also increases the release of inflammatory cytokines TNF-α and MCP-1 and upregulates T cells activation [26,27].

### 5.1. ACE Inhibitors (ACEi)

ACE inhibitors (ACEi) reduce plasma ATII concentrations and ATII-mediated vasoconstriction. This reduces pre- and afterload on the heart and decreases the risk of heart failure [58]. Blocking the pro-inflammatory effects of ATII with ACEi might improve the inflammatory environment in the heart [59]. Murine studies demonstrate that the ACEi enalapril reduced ATII-dependent monocyte recruitment from the spleen into the myocardium and increased ejection fraction by 14% [60]. Enalapril following MI also resulted in a significant reduction in the plasma levels of MCP-1 [61], a potent monocyte chemotactic agent [62].

### 5.2. Angiotensin II Type-1 Receptor Blockers (ARB)

Angiotensin II type-1 receptor blockers (ARB) block the AT_1_ receptor and similarly optimise cardiac output, and are often used when the side effects of ACEi cannot be tolerated [53]. ARB and ACEi have similar immunological effects, and clinical and preclinical studies have demonstrated that following MI, ARB reduce levels of pro-inflammatory cytokines IL-6 and TNF-α, while enhancing anti-inflammatory cytokine production [28,29]. ARB-mediated reduction of MCP-1 expression results in fewer monocytes and macrophages infiltrating the damaged myocardium, limiting cardiac remodelling and fibrosis [32]. Furthermore, ARB decrease the degree of ischaemic injury, infarct size, cardiomyocyte damage, and blood flow impairment [28]. A clinical trial comparing ramipril (ACEi) and olmesartan (ARB) showed a consistent reduction in infarct size, macrophage infiltration, and associated IL-1β and IL-6 levels for both dugs. However, olmesartan improved physiological parameters to a greater extent [30]. ARB also improve vascular inflammation by reducing oxidative stress, MCP-1 levels, macrophage and monocyte infiltration, vessel wall NADPH, and TNF-α in perivascular fat [31], and increase IL-10 levels [33]. 

### 5.3. Angiotensin Receptor–Neprilysin Inhibitors (ARNi)

The neutral endopeptidase neprilysin catalyses the degradation of atrial and brain natriuretic peptides (ANP, BNP), bradykinin and ATII. Inhibition of neprilysin results in natriuretic and antiproliferative effects and vasodilation [63]. The most prominent ARNi is entresto, a combination of sacubitril (neprilysin inhibitor) and valsartan (ARB), which is used as an alternative to or alongside ACEi and ARB in patients with symptomatic chronic heart failure with low ejection fraction [64]. The European Society of Cardiology HF guidelines recommend sacubitril/valsartan as a replacement for an ACEi to further reduce the risk of HF hospitalisation and death in ambulatory patients with HF with reduced ejection fraction (EF) who remain symptomatic despite optimal treatment with an ACEi, a β-blocker, and a mineralocorticoid receptor antagonists (MRA) [65]. Entresto decreases aldosterone release, fibrosis, and ventricular hypertrophy [66]. 

In experimental AMI, entresto suppressed pro-inflammatory cytokines IL-1β and IL-6 and extracellular matrix degradation by macrophages [34]. In diabetic mice with established heart failure, entresto reduced fibrosis by suppressing TGF-β [67]. 

### 5.4. Aldosterone Antagonists (AA)

Aldosterone antagonists (AA) reduce sodium and fluid retention, thus lowering blood volume and preload on the heart [53]. They dampen the detrimental effects of high plasma aldosterone concentrations post-MI, including progressive myocardial fibrosis, increased plasminogen activator inhibitor-1 (PAI-1) concentration, and decreased noradrenaline uptake [35]. Medical intervention is time-dependent, with optimal outcomes when administered within seven days after infarct [68]. Spironolactone, a competitive MRA, blocks the aldosterone receptors present on endothelial cells and monocytes, thus suppressing the downstream inflammatory cascade, which would otherwise lead to myofibroblast differentiation, collagen deposition, and fibrosis [36].

Fortuitous blocking of pro-inflammatory factors in the RAAS, thus, has immunomodulatory effects and may contribute to improved post-MI outcomes after treatment.

## 6. Conclusions and Future Perspectives

Immunological interventions are increasingly becoming integrated into everyday medical practice in various settings and are a promising expanding area of science. However, we still have a long way to go until a new immunomodulatory drug is used clinically in post-MI pharmacotherapy. Considering immunopharmacology of routine drugs, as well as their possible synergistic or antagonistic effects, with new therapies is a first step towards improved patient care and future trial design.

Clinical trials specifically targeting the post-MI immune response: A great number of clinical trials have aimed at immunomodulation post-MI, ranging from broad immunosuppressive approaches using corticosteroids, methotrexate or cyclosporine A, to more refined approaches targeting specific pathways and factors, including ROS, complement, mast cells, leukocyte infiltration, inflammatory cytokines IL-1, TNF-α and IL-6, and the inhibition of adaptive B and T lymphocytes [4]. The most promising target to date is IL-1. IL-1 inhibitors, including anakinra and canakinumab, which were initially developed for use in systemic inflammatory conditions, such as rheumatoid arthritis, have recently been tested in patients following AMI and show a significant benefit in the secondary prevention of subsequent cardiac events [69,70]. However, despite an enormous amount of effort and resources invested in these immunomodulatory trials, practical challenges, dealing with a highly diverse patient population, and striking heterogeneity in trial design mean that firm evidence in support of immunomodulatory treatment is still missing.

Therapeutic window: The post-MI immune response is highly dynamic and the pre-MI and the periprocedural clinical situation during PCI affect the degree of cardiac damage and therefore long-term complications, including the development towards HF. Some of the drug classes mentioned above are also administered perioperatively in patients undergoing PCI. 

Most prominently, antithrombotic agents, such as heparin and bivalrudin, are part of the acute pharmacological treatment regime of STEMI patients to restore blood flow. Heparin significantly reduced CRP levels when administered before reperfusion [71] and low-molecular-weight heparin (enoxaparin) significantly decreased IL-6 levels [72]. Bivalrudin, a direct thrombin inhibitor, is currently recommend by both EU and US guidelines for use during primary PCI instead of heparin plus platelet inhibitors [73]. Heparin has established anti-inflammatory effects [74], but studies on the immunological effects of bivalrudin are still missing. 

Another example, administration of the β-blocker metoprolol before reperfusion in patients undergoing PCI for STEMI, resulted in improved LVEF at six months follow-up, and a reduced occurrence of the prespecified composite of death, heart failure admission, re-infarction, and malignant arrhythmias at a median follow-up of two years, therefore indicating a possible long-term benefit of periprocedural β-blockers [75]. Immunological factors were not measured in this trial and it is therefore unclear if immunomodulation plays a role in these effects.

However, it is important to appreciate that any treatment to improve and accelerate reperfusion during AMI will affect the ensuing inflammatory response and downstream development to HF, which does not allow conclusions about specific immunomodulatory effects in a periprocedural setting.

New treatment strategies: The reality that despite potent post-MI pharmacotherapy, recurrent ischaemic events are common, drives the ongoing search for additional and better post-MI drugs. In particular, improved antithrombotic strategies seem warranted. Combinations of nonvitamin K antagonist oral anticoagulant (NOAC) with antiplatelet therapy have shown promising outcomes in large phase II and III randomised trials, suggesting that the use of NOAC in addition to standard antiplatelet therapy reduces the rate of recurrent ischaemic events. However, this comes at the price of increased risk for major bleeding [76]. 

Another recent addition to post-MI pharmacotherapy is ivabradine, a selective inhibitor of cardiac pacemaker cells in the sinus node of the right atrium, which controls the heart rate. Beyond its current use in treating stable angina [77], increasing evidence suggests ivabradine decreases cardiac structural remodelling and alleviates the risks of developing HF post-MI [78]. Immunological effects have been suggested by a study showing an increase in circulating numbers of myeloid dendritic cells (mDC) after six months of ivabradine treatment [78]. mDC negatively correlate with HF progression [79,80]. The same study showed a significant reduction in TNF-α concentrations [78]. A significant decrease in hsCRP levels was further recorded in patients treated with ivabradine for 30 days [81] which could be another plausible mechanism through which ivabradine reduces HF risk. In conclusion, despite a paucity of clinical trials, evidence suggests ivabradine has immunomodulatory effects.

Combination therapy: The immune response is a crucial player in post-MI restoration of tissue integrity. However, while immunomodulation may prevent ongoing damage, it is unlikely to replace tissue that has been lost already. Combining immunomodulation with regenerative therapies, such as biomaterial-based strategies or cell therapies aiming to replace damaged cardiomyocytes, seems the most promising future strategy [3]. Numerous studies have evaluated the effect of stem cell mobilisation, recruitment or transplantation into the myocardium [82,83]. While both fields are still in their infancy, once initial hurdles are overcome, the outlook for cardiac regeneration post-MI is promising.

Complications of immunotherapies: Potential side effects need to be carefully considered before the administration of immunosuppressive drugs post-MI. The immune system needs to be tightly regulated to ensure efficient responses against infections and cancers, while preventing autoreactivity. Suppressing the immune system post-MI may impact wound healing and regenerative processes, weaken defences against infectious diseases, and undermine the ability of the immune system to detect and destroy malignant growth early on. Chronic immunosuppressive treatment using methotrexate, cyclosporine or anti-TNF reagents is known to carry an increased risk of lymphoma, due to decreased immunosurveillance [84].

## Figures and Tables

**Table 1:** Post-myocardial-infarction (MI) and anti-heart-failure drugs and their immunomodulatory properties.

Drug Class		Example	Reported Immunological Effect
**Platelet inhibitor**	Cyclooxygenase enzyme inhibitors	Aspirin	Decrease ROS formation [6] Decrease leukocyte activation [6] Decrease hs-CRP [7] Decrease IL-6 [7]
	P2Y_12_ inhibitors	Clopidogrel Prasugrel Ticagrelor	Decrease hs-CRP [8] Decrease platelet degranulation [6] Decrease pro-inflammatory cytokines [6] Decrease leukocyte activation [6]
	GP IIb/IIIa inhibitors	Tirofiban	Decrease CRP levels [9]
**Statins**	HMG-CoA reductase and intracellular GTPase inhibitors	Atorvastatin Rosuvastatin Simvastatin	Decrease T cell activation [10,11,12,13] Increase FoxP3 in Treg cells [14,15,16] Decrease IL-2 and TNF-α [17]
**β-blockers**	Selective β1-blockers	Bisoprolol Nebivolol Metoprolol	Decrease TNF-α and restore cytokine network in DCM [18] Decrease TNF-α, IL-6, IL-10, sIL-2R, MCP-1, IL-18 in CHF [19] Decrease statin-mediated CRP decrease [20]
	Nonselective β-blockers	Propranolol	Decrease statin-mediated CRP decrease [20] Increases IL-1β and IL-6 6 h after MI [21] Increase NK cell activity [22]
	β1-β2-α-blockers	Carvedilol	Decrease HLA-DR^+^ and cytotoxic T-cell activation [23] Decrease ROS production [24] Decrease CCL2 [25]
**Drugs targeting the RAAS system**	Angiotensin converting enzyme inhibitors	Captopril Enalapril Ramipril	Decrease TNF-α and MCP-1 [26,27] Decrease T cell activation [26,27]
	Angiotensin receptor blockers	Azilsartan Candesartan Losartan	Decrease IL-6, TNF-α and IL-1β [28,29,30,31] Decrease oxidative stress [31] Decrease MCP-1 expression [31,32] Increase IL-10 levels [33]
	Angiotensin receptor–neprilysin inhibitors	Entresto (Sacubitril/Valsartan)	Decrease IL-6 and IL-1β [34] Decrease collagen deposition [34]
	Aldosterone antagonists	Eplerenone Spironolactone	Decrease PAI-1 levels [35] Decrease collagen deposition [36]

ROS, reactive oxidative species; hs-CRP, high sensitivity C-reactive protein; CRP, C-reactive protein; IL, interleukin; TNF-α, tumour necrosis factor-α; FoxP3, forkhead box P3; Treg, T regulatory cell; DCM, dilated cardiomyopathy; NK, natural killer cell; CCL2, chemokine ligand 2; HLA-DR, human leukocyte antigen–DR isotype; MCP, monocyte chemoattractant protein; PAI-1, plasminogen activator inhibitor-1; HMG-CoA, β-Hydroxy β-methylglutaryl-coenzyme A.
